# The Impact of Rapid Molecular Diagnostics for Influenza on Antibiotic Stewardship in the Emergency Department—An Observational Retrospective Study

**DOI:** 10.3390/antibiotics14020120

**Published:** 2025-01-23

**Authors:** Elisa Roth, Marco Cattaneo, Yvonne Hollenstein, Maja Weisser, Stefano Bassetti, Sarah Tschudin Sutter, Roland Bingisser, Christian H. Nickel, Adrian Egli

**Affiliations:** 1 Applied Microbiology Research, Department of Biomedicine, University of Basel, 4031 Basel, Switzerland; 2 Department of Clinical Research, University of Basel and University Hospital Basel, 4031 Basel, Switzerland; 3 Division of Infectiology and Hospital Hygiene, University of Basel and University Hospital Basel, 4031 Basel, Switzerland; 4 Division of Internal Medicine, University Hospital Basel, 4031 Basel, Switzerland; stefano.bassetti@usb.ch; 5 Emergency Department, University Hospital Basel, 4031 Basel, Switzerland; 6 Institute of Medical Microbiology, University of Zurich, 8006 Zurich, Switzerland

**Keywords:** influenza, rapid PCR, diagnostics, molecular diagnostics, antibiotic stewardship, management, respiratory tract infection

## Abstract

Objective: The clinical diagnosis of respiratory tract infections (RTIs) may result in unnecessary antibiotic treatment due to clinical exams’ low sensitivity and specificity to differentiate viral from bacterial infections and costly diagnostic work-ups. Unnecessary antibiotic consumption drives antibiotic resistance. We explored whether a rapid influenza-specific polymerase chain reaction (PCR) assay reduced antibiotic use in an emergency room before the COVID-19 pandemic. Methods: We conducted an observational retrospective study of patients with RTI symptoms treated in the ER of the University Hospital Basel from September 2014 to June 2015. We evaluated the impact of rapid diagnostic results, such as an influenza-specific PCR, blood sample results, and radiological imaging, on antibiotic prescription rates. Patient-related confounding factors were included since a patient’s clinical condition affects doctors’ clinical decision-making. Results: We included 607 patients with RTIs, tested with PCR for influenza A or B. Logistic regression showed that the odds ratio (OR) of being treated with antibiotics was significantly reduced by more than two-thirds in patients with a positive influenza PCR result (OR = 0.37; 95% CI, 0.22–0.59; *p* < 0.001). Increasing C-reactive protein (CRP) levels by tenfold (OR = 5.14; 95% CI, 3.34–8.12; *p* < 0.001) or suspected chest infection on a radiograph (OR = 5.81; 95% CI, 3.23–10.89; *p* < 0.001) increased the OR of antibiotic treatment by fivefold. The highest OR for antibiotic prescription was due to increased procalcitonin level by tenfold (OR = 10.13; 95% CI, 4.79–23.4; *p* < 0.001). Conclusions: Our study provides real-world evidence from a pre-COVID-19 ER setting of diagnostic tools used for RTI evaluation and their impact on antibiotic prescriptions. Rapid influenza-specific PCR results may affect the prescription rate of antibiotics but should be seen as part of a comprehensive diagnostic approach to guide clinical decision-making.

## 1. Introduction

The rapid identification of viral or bacterial pathogens is crucial for clinical decision-making and likely impacts the effective prescription of antibiotics. However, microbiological test results are often not available at the time of decision-making. Therefore, at the time given, it remains unclear if and which antibiotic drugs should be prescribed. The untargeted usage of antibiotics in outpatient and emergency consultations is a crucial contributor to the emergence of antimicrobial-resistant bacteria [1,2,3]. In the past decade, a global antibiotic resistance crisis has emerged. Data show that in 2019, 1.27 million (95% CI, 0.911–1.71) deaths were caused by antimicrobial resistance worldwide [4].

Viral respiratory tract infections (RTIs) during winter months are an essential factor contributing to antibiotic prescription. On a global scale, the impact is tremendous, with millions of infection episodes occurring annually [5]. This results in an enormous burden on the healthcare system every year. The highest proportion of emergency room (ER) consultations are due to community-acquired RTIs caused by both viral and bacterial pathogens, which also make up the leading group of infections treated with antibiotics [6,7]. However, there is a clinical challenge in assessing patients due to the high similarity of signs and symptoms shared by viral and bacterial RTIs, which makes a diagnosis based on clinical assessment alone very challenging. This can lead to the inappropriate prescription of antibiotics for viral-caused RTIs [8]. Efforts have been made to pursue antibiotic stewardship programs in ERs—several studies have indicated that rapid microbiological testing can reduce antibiotic prescription in ERs [6,7,8]. Therefore, we were curious to find out how implementing PCRs in tertiary hospitals in Switzerland affects the prescriptions of antibiotics and hence antibiotic stewardship.

Timely test results are crucial to support antibiotic stewardship. Culture-based microbiological diagnostics is time-consuming and often requires up to 72 h from sample collection to antibiotic susceptibility testing. The advantage of culture-based microbiology diagnostics is the identification of bacterial pathogens causing the RTI and adequate antibiotic susceptibility testing. However, in an outpatient and ER setting, the waiting time is too long to impact the prescription of antibiotics immediately. Various diagnostic tools, such as rapid tests based on the molecular or antigen detection of the pathogen or the host’s inflammatory response, promise rapid differentiation between a viral or bacterial pathogen within a few minutes to hours. Rapid results include laboratory parameters such as C-reactive protein (CRP), procalcitonin, or leucocyte counts.

Furthermore, chest radiographs are commonly used as a diagnostic approach in RTIs. Some studies have documented the value of such tests for antibiotic stewardship [9,10], whereas others have challenged the impact [11]. The effect of polymerase chain reaction (PCR) testing covering influenza and other respiratory viruses is enhanced by its short turnaround time (TAT) of two hours or less [12]. Three recent studies using panel PCR testing suggested that a diminished turnaround time allows for the faster adaption of treatment [12]. Broad panel PCRs often include more than 20 pathogens but are significantly more expensive and slower than narrow-targeted PCRs, including just three to four viral pathogens [13,14]. There has been no evidence of which rapid diagnostic test has the most significant influence on antibiotic prescribing. Our objective was to mitigate this critical gap in knowledge through the execution of a retrospective, observational study within a real-world context to explore the impact of multiple laboratory tests on antibiotic prescription rates in a pre-COVID-19 ER setting.

## 2. Results

### 2.1. Patient Characteristics

**Figure 1:**
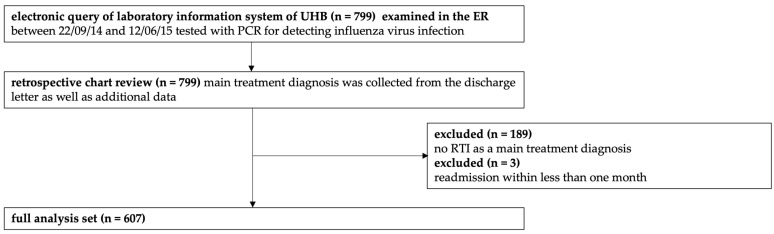
Study flowchart outlining patient inclusion and exclusion criteria. Abbreviations: University Hospital Basel, UHB; emergency room, ER; polymerase chain reaction, PCR; respiratory tract infection, RTI.

We included a total of 607 patients in the analysis. Figure 1 shows the selection of patients for the study. The suitable patients were identified via an electronic query of the laboratory information system of the UHB. The included patient samples were tested between 22 September 2014 and 12 June 2015 for influenza A and B via a nasal-pharyngeal swab. From this sample set, every discharge letter was reviewed for the primary treatment diagnosis. We identified 799 eligible patients via a query of the laboratory information system. One hundred eighty-nine patients being treated for other diseases than RTI were excluded. Three more patients were excluded due to the second exclusion criterion of being admitted to the ER within less than one month (Figure 1).

The median age of the examined cohort was 68.0 years (IQR 50.0, 80.0), with a marginal predominance of male patients (54.5%). Patients treated or prescribed with or without antibiotics were balanced with 320 (52.7%) and 287 (47.3%), respectively. Half the patients were influenza-positive, 301/607 (49.6%). Influenza A was detected in 31.8% (193/607) and influenza B in 17.8% (108/607) of patients. The median TAT for the PCR assay was 1.35 h (IQR 1.2, 1.6). All variables were chosen based on the accessibility of the results when the patient was still present in the ER.

### 2.2. Primary Endpoint Impact on the Antibiotic Prescription Rate

Table 1 demonstrates variances among patients receiving antibiotic treatment versus those without (Table 1). The univariate effect analysis of each covariable according to the prescription of antibiotics showed that patients who were treated with antibiotics had a higher median Charlson Comorbidity Index (CCI) compared to patients without antibiotic treatment (4, (IQR 2.0, 6.0) vs. 3, (IQR 0.0, 5.0); *p* < 0.001). In the group treated with antibiotics, the median leucocyte count was significantly higher compared to the patients without antibiotics (9.8 × 10^9^ cells/L, (IQR 7.4, 13.3) vs. 7.5 × 10^9^ cells/L (IQR 5.8, 9.9); *p* < 0.001). Median procalcitonin levels were almost three times higher within antibiotic-treated patients compared to patients without antibiotic treatment (0.23 ng/L, (IQR 0.11, 0.66) vs. 0.08 ng/L, (IQR 0.05, 0.12); *p* < 0.001), and the median CRP levels were four times higher (88.2 mg/L (IQR 40.0, 164.7) vs. 18.0 mg/L, (IQR 7.4, 41.0); *p* < 0.001).

Among patients treated or prescribed with an antibiotic, we found 115/320 (35.9%) were influenza-PCR-positive, whereas two-thirds (205/320, 64.1%) had an influenza-PCR-negative result. In the patient sample without antibiotic treatment, 186/287 (64.8%) were influenza-PCR-positive and one-third were influenza-PCR-negative (101/287, 35.2%).

### 2.3. Factors on Antibiotic Prescription

In the multivariable model, we found that all the assessed rapid diagnostic tests facilitating distinguishing between a viral or bacterial respiratory infection were associated with a difference in the prescription rate of antibiotics. Higher CRP and procalcitonin blood levels were associated with antibiotic treatment more frequently, while positive influenza PCR results were associated with lower antibiotic prescriptions. Of interest, previously reported positive associations such as age, gender, and leucocytes were not statistically significantly different in our multivariate model. Clinical symptoms did not statistically influence the decision to prescribe antibiotics.

**Figure 2:**
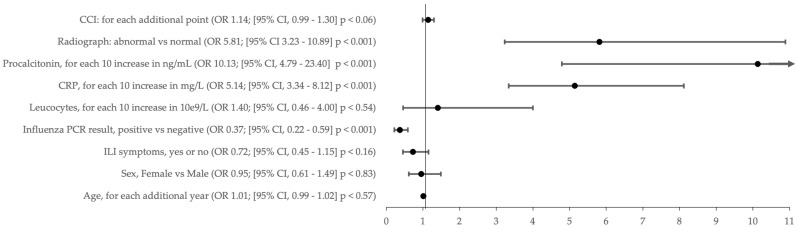
Odds ratios for antibiotics prescription. Odds ratio (OR) = •.

As shown in Figure 2, the odds ratio to prescribed antibiotics were increased with rising blood levels of CRP (OR 5.14; (95% CI 3.34–8.12)), increasing levels of procalcitonin (OR 10.13; (95% CI, 4.79–23.4)), and an abnormal finding on chest radiography (OR 5.81; (95% CI 3.23–10.89)). The OR of antibiotic treatment significantly decreased with a positive influenza result (OR 0.37; (95% CI, 0.22–0.59)). Age, sex, symptoms of ILI, increased leucocytes, and the CCI were not associated with any change in OR concerning the prescription of antibiotics (Figure 2).

## 3. Discussion

Utilizing a multivariate logistic regression model, we were able to show the association of rapid influenza-specific PCR with reduced antibiotic prescription. Contrary to this, elevated CRP, increasing procalcitonin levels, and abnormal chest radiographs were associated with increased odds ratios for antibiotic prescription. Leucocytes did not impact antibiotic prescriptions in the multivariate analysis, which is unsurprising due to their low specificity in acute respiratory disease [15]. Laboratory testing positively impacted antibiotic stewardship, whereas clinical examination alone could not separate bacterial and viral infections.

Antimicrobial stewardship is a cornerstone of reducing the burden of antimicrobial resistance, and the proper diagnostic work-up of bacterial and viral infections is critical. Therefore, these minimally invasive tests should be considered as tools to reduce antibiotic over-prescriptions in an ER setting. Additionally, recent studies have shown that point-of-care influenza testing is cost-effective in the emergency department setting [16].

Prompt and adequate decision-making in the ER is critical in this commonly overcrowded setting. Prior studies have shown that applying rapid influenza-specific PCR testing can streamline testing and improve antiviral stewardship and ER workflow, including reducing the length of stay at the ER [17]. Even though this study was performed before the COVID-19 pandemic, separating viral and bacterial infections remains an important aspect of rapid and effective patient management.

Our primary goal was to highlight the value of rapid influenza-specific PCR diagnostics, as evidence surrounding the usefulness of this test to decrease antibiotic prescription is still scarce. The OR was significantly lower in patients with a positive PCR result for either influenza A or B. However, the univariate analysis showed that 35.9% of the influenza-positive patients received antibiotics. This indicates that clinical decision-making does not rely on one specific test but is usually built on multiple factors, probably also on the “gut feeling” of the clinician. Why patients with a confirmed viral infection still receive antibiotic treatment should be evaluated in detail in further studies. Our findings align with other studies, demonstrating the impact of timely available PCR results on the antibiotic prescription pattern of doctors in the ER [18]. This contrasts with previously published studies that remained inconclusive about the effects of different rapid testing results on reducing antibiotic prescriptions [5,6,7]. Furthermore, we found that elevated levels of procalcitonin and CRP were correlated with an increased odds ratio (OR) for antibiotic prescriptions.

As a retrospective single-center study, our work has significant limitations: The most limiting factor was that we did not use current data. Still, it is interesting to see retrospectively how the implementation of PCRs has changed over time. Second, retrospective studies always show a particular bias regarding the included population, as we cannot guarantee that the examined diagnostic tests were used entirely unbiasedly. Indeed, in the ER, some algorithms determine when to use which tests to make a clinical decision process efficient. Clearly, during and since the COVID-19 pandemic, the usage of various virus-specific PCR diagnostics in ER settings has amplified. Third, our study cannot make any conclusions regarding the appropriateness of the initiated antibiotic therapy since respiratory bacterial infectious diseases are difficult to prove in the ER setting. This is a critical aspect of this type of study. What is the reference standard to establish whether an RTI was present? In our case, we have chosen the diagnosis carried out by the ER team. Ideally, a panel of independent physicians would prospectively integrate all information and assess each case. Broad-panel PCRs could add valuable information about either atypical bacterial infection or a more comprehensive range of viruses [19]. Still, these panel PCRs are too expensive and not commonly used in the outpatient setting.

Future studies should evaluate the impact of new panel PCRs to determine and separate bacterial and viral pathogens for antibiotic stewardship. Furthermore, we suggest systematically identifying the different antimicrobials to better understand the treatment choice. However, looking into subgroups of antibiotics increases the heterogeneity and requires larger study cohorts. Our results show that rapid diagnostic testing, including influenza-specific PCR and inflammatory parameters, may be a critical diagnostic step to decrease antibiotic prescription. Our study highlights the need to conduct prospective, randomized, controlled studies to further investigate the effectiveness of rapid diagnostic testing in antibiotic stewardship.

## 4. Methods

### 4.1. Study Design

We performed a retrospective, observational cohort study at the University Hospital Basel (UHB), a 700-bed tertiary medical center with about 45,000 ER visits in 2015. We included adult patients aged over 18 years who were evaluated with an influenza-specific rapid PCR (GeneXpert, Cepheid, Sunnyvale, CA, USA). Patient charts were retrospectively analyzed.

### 4.2. Aims

We aimed to assess the effect of different rapid-access diagnostic tests on the prescription rate of antibiotics in patients with acute RTIs presenting to the ER. We considered different rapidly available diagnostic parameters compared to each other to examine which one has the highest impact on clinical decision-making in this specific ER setting.

### 4.3. Ethics

The ethical proposal for this study follows Swiss laws. Switzerland follows the declaration of Helsinki, and our proposal has been evaluated and approved by the Ethics Committee for Northern and Central Switzerland (EKNZ) (ref. nr. 2015-363 and 2019-00802).

The ethics committee waived the requirement for informed consent based on Swiss regulations for the further use of biological material and health-related personal data without new consent. This decision is governed by Article 34 of the Human Research Act (HRA) and Articles 37–40 of the Human Research Ordinance (HRO).

### 4.4. Recruitment and Inclusion/Exclusion Criteria

Patients were included (i) if they received a rapid PCR (GeneXpert, Cepheid, Sunnyvale, CA, USA) ordered within the first 24 h of their ER stay and the results were either influenza A or influenza B positive or negative and (ii) if the primary diagnosis of their ER admission was an acute respiratory illness. The data included were collected retrospectively between 9 September 2014 and 12 June 2015. Clinical practice guidelines define an acute respiratory illness with signs and symptoms of upper or lower RTI with or without fever [20]. The influenza-specific PCR result was usually available during the time in the ER. Patients were excluded if their primary working diagnosis was not an RTI and if they were repeatedly admitted during the last month.

### 4.5. Data Collection and Definitions

Data access was restricted to the authors involved in data collection to maintain participant confidentiality throughout and after the study. For each included patient, the following data were collected: age (in years), gender (male/female/unknown), and the Charlson Comorbidity Index (CCI, including the following comorbid conditions: age, myocardial infarction, chronic heart failure, peripheral vascular disease, cerebrovascular accident or transient ischemic attacks, dementia, chronic obstructive pulmonary disease (COPD), connective tissue disease, liver disease, diabetes mellitus, hemiplegia, moderate (creatinine > 0.27 mmol/L) to severe chronic kidney disease (dialysis or post kidney transplant), solid tumor, leukemia, lymphoma, and AIDS) [21] according the medical report at the time of discharge.

Furthermore, we searched for symptoms of influenza-like illness (ILI), such as fever and acute cough (lasting less than ten days) [22] before or during the ER admission.

If the period of the symptoms was not indicated, it was assumed to be fewer than ten days. When ILI symptoms were not mentioned in the discharge letter, they were considered non-existent. Furthermore, relevant laboratory parameters (e.g., white blood cell count, CRP, and procalcitonin; continuous variables), influenza-specific PCR result (detection/no detection of influenza A or B), primary treatment diagnosis (RTI versus other), and receipt of antibiotics or lack thereof when the treatment was started in the ER were all included. Chest radiographs obtained within 24 h of admission were classified as abnormal if pneumonia was suspected.

### 4.6. Statistical Analysis

The study’s primary endpoint was the administration or prescription of antibiotic treatment in the ER. All analyses were conducted with the statistical software package R, using “two-sided’‘ statistical tests and confidence intervals with standard significance and confidence levels α = 5% and (100% − α) = 95%, respectively. Missing values were ignored in the descriptive analyses—the patient characteristics and numbers of missing values are illustrated in Table 1. We used a logistic regression model to compare the odds ratio of impact, assessing whether variables had an increasing or decreasing effect compared to each other. All classical laboratory parameters were available within three hours.

We performed a multivariable logistic regression for antibiotic prescription, including different covariates. The missing values of the covariates with less than 1% missing values (CRP and leucocytes) were imputed as the medians of the corresponding non-missing values. For covariates with missing values of more than 20% for each categorical covariate, an additional category was introduced for missing values, while for each numerical covariate, a dummy variable was introduced, indicating whether the value was missing or not. The Holm–Bonferroni method was used to control the family-wise error rate. We reported *n* (%) for categorical variables and median (range) for continuous variables and interquartile ranges (IQRs), with *p*-values for the effect of the grouping obtained by Pearson’s chi-squared test and by Kruskal–Wallis one-way analysis of variance, respectively. A *p*-value < 0.05 was statistically significant.

We measured *n* (%) for categorical variables and medians for continuous variables with interquartile range (IQR) and *p*-values for the effect of the grouping obtained by Pearson’s chi-squared test and Student’s *t*-test, respectively.

## 5. Conclusions

Our study demonstrates the association of rapid diagnostic results, particularly positive influenza A or B PCR results, with reduced antibiotic prescriptions in an ER setting. It highlights the potential of PCR in clinical decision-making and antibiotic stewardship while using minimally invasive tests. Further prospective studies are needed to assess the clinical impact and cost-efficiency of such diagnostics in clinical management.

## Figures and Tables

**Table 1:** Baseline characteristics and univariate analysis.

	Total Cohort *n* = 607 Patients	% Missing	Patients with Antibiotics Prescribed *n* = 320 (52.7%)	Patients with No Antibiotics Prescribed *n* = 287 (47.3%)	*p* Value
Patient demographics					
Age	68.0 (50.0, 80.0)	0.0	72 (55.0, 82.0)	62.0 (44.5, 78.5)	<0.001
Sex		0.0			0.014
Male	331 (54.5%)		190 (59.4%)	141 (49.1%)	
Female	276 (45.5%)		130 (40.6%)	146 (50.9%)	
Rapidly available microbiological relevant results					
Influenza PCR positive	301 (49.6%)	0.0	115 (35.9%)	186 (64.8%)	<0.001
Leucocytes 10^9^/L cells/L	8.7 (6.4, 11.8)	0.2	9.8 (7.4, 13.3)	7.5 (5.8, 9.9)	<0.001
CRP mg/L	44.0 (14.4, 101.7)	0.2	88.2 (40, 164.7)	18 (7.4, 41)	<0.001
Procalcitonin ng/L	0.13 (0.07, 0.3)	23.2	0.23 (0.11, 0.66)	0.08 (0.05, 0.12)	<0.001
Radiographic signs of pulmonary infections Present	160 (34.6%)	23.7	139 (52.7%)	21 (10.6%)	<0.001
CCI	4.0 (1.0, 5.0)	0.0	4.0 (2.0, 6.0)	3.0 (0.0, 5.0)	<0.001
ILI symptoms present	248 (40.9%)	0.0	121 (37.8%)	127 (44.3%)	0.126

Abbreviations: University Polymerase chain reaction, PCR; C-reactive protein, CRP; Charlson Comorbidity Index, CCI; influenza-like illness, ILI.

## Data Availability

Raw data supporting our conclusions will be made available upon request.
